# Bacterial Inhibition on *Beauveria bassiana* Contributes to Microbiota Stability in *Delia antiqua*

**DOI:** 10.3389/fmicb.2021.710800

**Published:** 2021-10-06

**Authors:** Fangyuan Zhou, Yunxiao Gao, Mei Liu, Letian Xu, Xiaoqing Wu, Xiaoyan Zhao, Xinjian Zhang

**Affiliations:** ^1^Shandong Provincial Key Laboratory of Applied Microbiology, Ecology Institute, Qilu University of Technology (Shandong Academy of Sciences), Ji'nan, China; ^2^State Key Laboratory of Biocatalysis and Enzyme Engineering, School of Life Sciences, Hubei University, Wuhan, China

**Keywords:** animal-microbe symbiosis, antifungal, defensive association, onion maggot, insect-microbe

## Abstract

Given the multiple roles of associated microbiota in improving animal host fitness in a microbial environment, increasing numbers of researchers have focused on how the associated microbiota keeps stable under complex environmental factors, especially some biological ones. Recent studies show that associated microbiota interacts with pathogenic microbes. However, whether and how the interaction would influence microbiota stability is limitedly investigated. Based on the interaction among *Delia antiqua*, its associated microbiota, and one pathogen *Beauveria bassiana*, the associated microbiota's response to the pathogen was determined in this study. Besides, the underlying mechanism for the response was also preliminarily investigated. Results showed that *B. bassiana* neither infect *D. antiqua* larvae nor did it colonize inside the associated microbiota, and both the bacterial and fungal microbiota kept stable during the interaction. Further experiments showed that bacterial microbiota almost completely inhibited conidial germination and mycelial growth of *B. bassiana* during its invasion, while fungal microbiota did not inhibit conidial germination and mycelial growth of *B. bassiana*. According to the above results, individual dominant bacterial species were isolated, and their inhibition on conidial germination and mycelial growth of *B. bassiana* was reconfirmed. Thus, these results indicated that bacterial instead of fungal microbiota blocked *B. bassiana* conidia and stabilized the associated microbiota of *D. antiqua* larvae during *B. bassiana* invasion. The findings deepened the understanding of the role of associated microbiota–pathogen microbe interaction in maintaining microbiota stability. They may also contribute to the development of novel biological control agents and pest management strategies.

## Introduction

Microbes inhabiting on body surface, inside the guts, and in cells of animals establish stable microbial communities and form a close symbiotic association with the animal hosts. They contribute to nutritional provision (Douglas et al., 2001; Zhou et al., 2017; Noman et al., 2020; Cao et al., 2021; Luo et al., 2021), protection of the hosts against natural enemies (Kaltenpoth et al., 2005; Koch and Schmid-Hempel, 2011), detoxification of toxic chemicals in food (Shen and Dowd, 1991; He et al., 2018; Akami et al., 2019), regulation of developmental processes (Lee et al., 2019), and also mediate host behaviors (Douglas, 2015; Schneider et al., 2019). Dysbiosis of associated microbiota is fatal for animal hosts. For example, dysbiosis of gut microbiota accelerates the host's mortality when challenged by pathogens (Wei et al., 2017; Xu et al., 2019). Thus, increasing numbers of researchers have focused on how the associated microbiota keeps stable under complex environmental factors, especially some biological ones, which tries to illustrate the foundation of microbiota's multifunction.

Factors that can influence the stability of associated microbiota in animals mainly include host immunity (McMillan and Adamo, 2020), host diet (Tragust et al., 2020; Pan et al., 2021), and inhabited environment (Kudo et al., 2019; Cini et al., 2020; Nishino et al., 2021). In addition, some environmental microbes, especially animal pathogens, interact with the associated microbiota. For example, the gut bacterium *Lactobacillus kunkeei* in honey bee shows significant inhibitory effects on the pathogen *Paenibacillus* (Arredondo et al., 2018). The gut bacteria in scarab greatly inhibit the pathogen *Bacillus thuringiensis* (Shan et al., 2014). The symbiont-derived microbial protection for animal hosts against entomopathogenic fungi has also been widely reported in other taxonomic insect clades including bark beetles (Hulcr et al., 2011) and termites (Seipke et al., 2012). Although the interaction between the microbiota and pathogenic species has been frequently investigated, whether and how this interaction would influence microbiota stability remains unclear. Only a few studies have investigated the influence of the interaction between the associated microbiota and exogenous environmental factors such as pathogens on the stability of associated microbiota (Lee et al., 2013; Shao et al., 2017).

Dipterans host a myriad of microbes that facilitate their fitness (Ben-Yosef et al., 2014; Mazzetto et al., 2016; Zhou et al., 2019). In the symbiotic system formed by one such dipteran, *Delia antiqua*, and its associated microbes, several bacterial species are efficient protectors as they repress larval infection by *Beauveria bassiana* (Zhou et al., 2019, 2020). All these bacterial strains show significant inhibition on the conidial germination and mycelial growth of *B. bassiana*, which leads to the failure of the fungus to infect *D. antiqua* larvae. However, whether the microbiota associated with *D. antiqua* would be affected by *B. bassiana* is unknown. Reasonably, the symbiotic system formed by *D. antiqua* and its associated microbiota provides a good model to investigate the maintenance of associated microbiota. With this symbiotic system, we aimed to determine the response of associated microbiota in *D. antiqua* to pathogen invasion. Specifically, we first determined if the associated microbiota of *D. antiqua* larvae interacts with *B. bassiana*, i.e., whether the microbiota affects the colonization of the entomopathogen in the larvae and influences larval survival. Second, responses of the microbiota (including both the bacterial and fungal microbiota) to *B. bassiana* invasion were determined by comparing the microbiota associated with *D. antiqua* larva treated with/without *B. bassiana* using high throughput sequencing technology. Third, the influence of the bacterial and fungal microbiota on *B. bassiana* conidial germination and mycelial growth were determined. Fourth, according to the results of high throughput sequencing, dominant bacteria were isolated, and their specific effects on mycelial growth and conidial germination of *B. bassiana* were evaluated to confirm their contribution to the stability of the microbiota. The findings of this study deepened the understanding of the role of interaction of associated microbiota with pathogenic microbe in the maintenance of microbiota stability. The explanation as to why some insect larval stages are not prone to infection by some entomopathogenic fungi was also inferred and discussed. Thus, the results can provide a supporting base for the development of novel biological control agents and pest management strategies.

## Materials and Methods

### Insects and Microbial Strains

Non-axenic *D. antiqua* larvae were originally collected from garlic fields in Fanzhen (N36°14′, E117°25′), China in 2020. Axenic larvae were obtained by rearing surface-sterilized eggs (washed twice with 75% ethanol for 30 s) with an antibiotic-containing artificial diet (Zhou et al., 2019). Detailed information about artificial diets is provided in Supplementary Table 1. Further microbial isolation with Luria-Bertani agar (LBA) and potato dextrose agar (PDA) was conducted to ensure that all culturable bacteria and fungi associated with larvae were eliminated. As this insect is not in the state's conservation list of wild animals in China, collection permissions were not required. The fungal strain *B. bassiana* BB1101 was originally isolated from infected *D. antiqua* adults collected in fields (Zhou et al., 2019) and was preserved in the laboratory. Bacterial strains including *Empedobacter brevis* AB10, *Providencia burhodogranariea* CB11, *Lactococcus garvieae* FA27, *Acinetobacter johnsonii* MF17, *Acinetobacter guillouiae* MF06, and *Enterococcus saccharolyticus* FG16 were isolated from field-collected *D. antiqua* larvae by the previously described method of Zhou et al. (2019). Detailed information on the isolation and identification of these strains is provided in the Supplementary Material.

### Experiment I: Effects of *B. bassiana* BB1101 on *D. antiqua* Survival and *B. bassiana* BB1101 Colonization in *D. antiqua* Larvae

Larvicidal effects of *B. bassiana* BB1101 on both non-axenic and axenic *D. antiqua* larvae were determined. Specifically, 120 2nd instar non-axenic larvae of *D. antiqua* collected from 12 garlic plants in fields (10 larvae per garlic plant) were randomly assigned to two groups. In one group, the larvae were sprayed individually with conidia suspension from *B. bassiana* BB1101 (10^9^ conidia/ml) and air dried on a piece of sterilized filter paper (non-axenic + Bb). In the second group, the larvae were individually sprayed with sterilized phosphate-buffered saline (PBS) (non-axenic). The 2nd instar axenic larvae (obtained by rearing surface-sterilized eggs on artificial diets for 7 days; 60 larvae per group) were also divided into two groups and treated as described above (i.e., axenic + Bb, axenic, respectively). Then, larvae from the above-treated groups (non-axenic, axenic, non-axenic + Bb, and axenic + Bb) were separately fed on sterilized artificial diets without antibiotics and reared in dark at 24°C. Larval survival was monitored every 2 days until pupation. This experiment was repeated thrice.

To determine colonization of the fungus on *D. antiqua* larvae, *B. bassiana* were isolated. Specifically, the two groups of 2nd instar larvae including 30 non-axenic and 30 axenic larvae were treated with a conidia suspension of *B. bassiana* BB1101 (10^9^ conidia/ml) as described above. PBS was sprayed on the other two groups of 2nd instar larvae (30 non-axenic and 30 axenic larvae). Then, larvae from the above treatment groups (non-axenic-T, axenic-T, non-axenic-C, and axenic-C) were separately fed on sterilized artificial diets without antibiotics and reared in darkness at 24°C. Seven days later, *B. bassiana* were isolated from body surfaces and guts of the larvae (*n* = 30) according to a previously described method with minor modification (Zhou et al., 2019). PDA containing 10 mg/ml of penicillin and 10 mg/ml of streptomycin sulfate instead of LBA was used in this experiment. The number of *B. bassiana* that could be isolated from the body surface or gut of each larva was counted and recorded.

### Experiment II: Effects of *B. bassiana* BB1101 on the Microbiota of *D. antiqua* Larvae

#### *B. bassiana* BB1101 Larval Treatment and Microbiota Sample Collection

To determine the larval microbiota response to *B. bassiana*, a conidial suspension of the fungal strain BB1101 was used to treat the larvae. The samples were collected from both the larval body surfaces and guts and sequenced; the microbiota between treated and untreated larvae was compared. Briefly, for the treatment group, one 2nd instar larva was treated as described in experiment I. Subsequently, the larva was reared on surface-sterilized garlic pieces (twice with 75% ethanol for 30 s) inside a 30-mm Petri dish. Seven days later, the larva was put into a 1.5-ml Eppendorf tube containing 200 μl sterilized 1 × PBS solution, sonicated for 1 min, and vortexed for 30 s. The washing fluid was collected as the sample for body surface microbiota. The washed larva was surface-sterilized (twice with 75% ethanol for 30 s) and washed thrice with sterilized 1 × PBS; subsequently, the gut was dissected out, and the gut microbiota sample was collected. Sterilized PBS was used instead of the conidial suspension in the control group. In total, 20 larvae collected from 20 garlic plants in the fields (one larva per plant) were used in this experiment, and these larvae were randomly assigned to the control and treatment groups (*n* = 10 for each group).

#### DNA Extraction, PCR, Illumina MiSeq, and Sequencing Data Analysis of Microbiota Samples

DNA was extracted from the larval body surface and gut microbiota samples using the TIANamp DNA kit (TIANGEN Biotech Co. Ltd., Beijing, China). For detection of bacterial microbiota, the V3–V4 region of the 16S rRNA gene was amplified by PCR with the following primers sequences: 338F (5′- ACTCCTACGGGAGGCAGCAG-3′) and 806R (5′- GGACTACHVGGGTWTCTAAT-3′) (Xu et al., 2018a). The PCR mixture (20 μl) consisted of 10 ng DNA template, 10 μM primers (1 μl each), 2 μl 2.5 mM dNTPs, 0.3 μl FastPfu Polymerase (Transgene, Beijing, China), and 4 μl 5 × FastPfu buffer. The PCR cycling program was as follows: 95°C for 10 min; 30 cycles of 30 s at 95°C, 55°C for 30 s, and 72°C for 45 s; and 72°C for 10 min. For detection of fungal microbiota, the following primers were used to amplify the ITS rRNA genes (Liu et al., 2018): ITS1F (5′-CTTGGTCATTTAGAGGAAGTAA-3′) and ITS2R (5′-GCTGCGTTCTTCATCGATGC-3′). The PCR reaction mixture (20 μl) consisted of 10 ng template DNA, 0.8 μl of each primer (5 μM), 2 μl of 2.5 mM dNTPs, 4 μl of 5 × FastPfu Buffer, 0.4 μl of FastPfu Polymerase (Transgene, Beijing, China), and 0.2 μl bovine serum albumin (BSA). The PCR cycling program was as follows: 95°C for 3 min, 35 cycles of 30 s at 95, 55°C for 30 s, 72°C for 45 s, and 72°C for 10 min. For each DNA sample, the PCR was conducted in three technical replicates and then the PCR products were mixed. Subsequently, the PCR products were verified using a 2% agarose gel, extracted from the gel, and purified using the AxyPrep DNA Gel Extraction Kit (Axygen Biosciences, Union City, CA, United States). The purified PCR products were quantified using QuantiFluor^TM^-ST (Promega, Madison, WI, United States). Finally, the PCR products were “paired-end” sequenced (2 × 300) on the Illumina MiSeq PE300 platform (Illumina, San Diego, CA, United States).

Obtained sequences were assigned to samples according to specific barcodes. The barcodes and the primers were subsequently removed. The paired-end reads were assembled using FLASH V1.2.71 (Magoc and Salzberg, 2011). Low-quality data were filtered by QIIME 1.9.0 (Quantitative Insights into Microbial Ecology) software package with default parameters (Caporaso et al., 2010), and chimeric sequences were removed using UCHIME algorithm (Edgar et al., 2011). Effective reads from each sample were initially clustered into operational taxonomic units (OTUs) with 97% similarity by UPARSE pipeline (Edgar, 2013). The most abundant sequence for each OTU was selected as the representative sequence (DeSantis et al., 2006) and annotated by the RDP classifier according to the SILVA and the UNITE databases with a confidence threshold of 70% (Quast et al., 2013). All sequence data were deposited in GenBank (PRJNA756127).

Rarefaction curves were estimated with the “alpha_rarefaction.py” script at the 97% similarity and a cutoff of 43,000. Alpha diversity indices (including Simpson, Shannon, Chao1, and ACE indices) were calculated with the “alpha_diversity.py” script. Diversity indices and the relative abundance of different genera in the control and *B. bassiana*-treated groups were compared using Mann–Whitney *U* test. Non-metric multidimensional scaling (NMDS) was used to evaluate the sample cluster based on Bray–Curtis similarity. Composition differences were determined by analysis of similarities (ANOSIM) with 999 permutations using PAST (Hammer et al., 2001). Weighted Unifrac principal coordinate analysis (PCoA) with Fast Unifrac (Hamady et al., 2010) was used to identify the sample clusters. Besides, a permutational multivariate analysis of variance (PERMANOVA) based on the weighted UniFrac distance (999 permutations) was used to determine differences in community composition between the control group and the *B. bassiana*-treated groups (Chen, 2012). The genus abundance between the two groups was compared using the Student's *t*-test.

### Experiment III: Effects of Bacterial and Fungal Microbiota on Conidial Germination and Mycelial Growth of *B. bassiana* BB1101

#### Bacterial, Fungal, and Limited Microbiota Collection

Larval bacterial, fungal, and limited (both bacteria and fungi were partly removed) microbiota were obtained by antibiotic treatment according to a previously described protocol (Cheng et al., 2018).

Bacterial microbiota was obtained from six larvae collected from six garlic plants (one larva per plant, *n* = 6) in fields. To obtain bacterial microbiota from the larval body surface, each larva in 900 μl sterilized 1 × PBS solution was sonicated for 1 min and vortexed for 30 s. The washing fluid was collected in two 1.5-ml Eppendorf tubes (400 μl for each tube). Subsequently, 400 μl of an antibiotic stock solution containing 10 mg/ml of nystatin and 10 mg/ml of cycloheximide was added into one of the tubes and 400 μl of sterilized water to the other (control). The two tubes were incubated at 4°C for 12 h. Subsequently, 100 μl of the solution from each of the two tubes was diluted in 1 × PBS and spread on Luria-Bertani (LB) and potato dextrose broth (PDB) agar plates. Single colonies (identified initially by morphology) were collected, streaked thrice, and determined either as bacteria or fungi by PCR amplification of 16S and ITS rRNA gene as described in *Insects and Microbial Strains* section, respectively. The number of isolates was counted. The remaining 300 μl of the solution was used as a bacterial microbiota sample from the larval body surface. The corresponding larvae from the above treatment were surface-sterilized and dissected to obtain the gut sample. The gut samples were transferred into new 1.5-ml Eppendorf tubes containing 900 μl of sterilized PBS solution; samples were ground and treated as described above to obtain bacterial microbiota samples from larval guts.

To obtain fungal microbiota from the larval body surface and gut samples, a similar treatment regime as described above was followed using an antibiotic stock solution containing 10 mg/ml of penicillin and 10 mg/ml of streptomycin sulfate (*n* = 6).

In addition, six larvae were treated with an antibiotic stock solution containing 10 mg/ml of nystatin, 10 mg/ml of cycloheximide, 10 mg/ml of penicillin, and 10 mg/ml of streptomycin sulfate as described above to eliminate both bacterial and fungal microbiota. After antibiotic treatment, only three bacterial species from the larval body surface microbiota belonging to *Xanthomonas* and *Bacillus* were isolated and identified; these were termed as “limited microbiota of larval body surface” (*n* = 6). Neither bacterial nor fungal isolates were isolated from the gut microbiota samples after the antibiotic treatment, and these were termed as “limited microbiota of larval gut” (*n* = 6); PDB was used as the media.

#### Effects of Bacterial, Fungal, and Limited Microbiota on Conidial Germination of *B. bassiana* BB1101

To determine the effects of bacterial microbiota obtained from larval body surface on conidial germination of *B. bassiana*, 300 μl of the bacterial microbiota samples from larval body surface obtained from the method described above were centrifuged at 3,000*g* for 5 min. The precipitate was washed thrice with 1 × PBS and suspended in 2 ml of LB media; it was then transferred into a new test tube and incubated at 28°C, 180 rpm for 72 h. The supernatant was collected by centrifuging the culture at 6,000*g* for 5 min, and it was diluted with LB to 1, 5, and 25 times. In each of the diluted supernatants, 100 μl of conidial suspension of *B. bassiana* BB1101 [10^6^ colony forming units (CFU)/ml] was added to 3.9 ml of the supernatant. LB was used instead of the supernatant in the control group. The mixture was incubated at 25°C, 200 rpm for 24 h. Conidia were defined as germinated when the length of the germ tube was greater or equal to the conidia's under a microscope (Dantigny et al., 2006). In this experiment, six larvae (one larva per garlic plant) collected from the fields were used to obtain bacterial microbiota samples from larval body surface as described above. The experiment was repeated six times. The effects of bacterial microbiota from the larval gut on conidial germination of *B. bassiana* were similarly determined (*n* = 6).

The effects of fungal microbiota from larval body surface (*n* = 6) and gut (*n* = 6) samples on conidial germination of *B. bassiana* were also determined according to the methods described above. PDB was used instead of LB to prepare the fungal culture supernatant in the treatment group.

The effects of limited microbiota from larval body surface (*n* = 6) and gut (*n* = 6) samples on conidial germination of *B. bassiana* were also determined according to the method described above. After treatment with the antibiotic stock solution containing nystatin, cycloheximide, penicillin, and streptomycin sulfate, only three bacterial species from the larval body surface microbiota survived; LB media was used in the microbial culture supernatant. In the gut, both fungal and bacterial microbiota were eliminated by antibiotic treatment. Thus, PDB was used instead of the microbial culture supernatant to determine its effect on conidial germination.

#### Effects of Bacterial, Fungal, and Limited Microbiota on Mycelial Growth of *B. bassiana* BB1101

To determine the effects of bacterial microbiota from larval body surface on mycelial growth of *B. bassiana*, 300 μl of bacterial microbiota samples obtained from larval body surface sample was centrifuged at 6,000*g* for 5 min; the precipitate was washed thrice with 1 × PBS; suspended in 1 ml 1 × PBS; and diluted to 5 × 10^3^, 1 × 10^3^, and 2 × 10^2^ CFU/ml. Into 2 ml of each diluted microbiota sample, 18 ml of melted PDA was added, mixed, and poured into a 90-mm Petri dish. These plates were used in the treatment groups. For the control group, 2 ml of 1 × PBS was used instead of the microbiota suspensions. Agar plugs (3 mm) taken from the leading edge of the *B. bassiana* BB1101 PDA plates were inoculated at the center of the above PDA plates. Agar plugs from the same plates were randomly assigned to different treatment groups. These plates were subsequently incubated in dark at 25°C. The mycelial diameter was measured each day in two directions at right angles to each other during the experimental period. The mycelial growth was calculated as the percent value relative to that of the control. For the above experiment, six field-collected larvae (one larva per garlic plant) were used to obtain bacterial microbiota samples from the larval body surface. The experiment was repeated six times. The effects of bacterial microbiota from the larval gut on mycelial growth of *B. bassiana* were similarly determined (*n* = 6).

The effects of fungal microbiota from larval body surface (*n* = 6) and gut (*n* = 6) samples on mycelial growth of *B. bassiana* were also determined similarly as for bacterial microbiota.

The effects of limited microbiota from larval body surface (*n* = 6) and gut (*n* = 6) samples on mycelial growth of *B. bassiana* were also determined similarly as for bacterial microbiota. After treatment with the antibiotic stock solution containing nystatin, cycloheximide, penicillin, and streptomycin sulfate, only three bacterial species of the larval body surface microbiota survived. In the gut, both fungal and bacterial microbiota were eliminated by antibiotic treatment. PDB was used instead of the microbiota to determine the effect on mycelial growth.

### Experiment IV: Effects of Dominant Bacterial Symbionts on Conidial Germination and Mycelial Growth of *B. bassiana* BB1101

The effects of dominant bacterial species including *E. brevis* AB10, *P. burhodogranariea* CB11, *L. garvieae* FA27, *A. johnsonii* MF17, *A. guillouiae* MF06, and *E. saccharolyticus* FG16 on conidial germination and mycelial growth of *B. bassiana* BB1101 were determined as described in experiment III with some minor modifications. Each bacterial strain was used instead of the microbiota (in experiment III) to determine the effects of the above bacterial strains on conidial germination of *B. bassiana* BB1101; LB was used as the control. A PBS suspension of cells from each bacterial strain was used instead of the microbiota (in experiment III) to determine its effect on the mycelial growth of *B. bassiana* BB1101; PBS was used as the control. Both the experiments were repeated six times (*n* = 6).

### Statistical Analysis

Prior to statistical analysis, Kolmogorov–Smirnov test and Levene's test were conducted to test the normality and homogeneity of all variances, respectively. Larval survival of different treatment groups in experiment I was compared using Kaplan–Meier analysis (log-rank test). The conidial germination or mycelial growth rate of *B. bassiana* BB1101 in experiments III and IV was compared with one-way or Welch' s ANOVA followed by Tukey or Dunnett's T3 multiple comparisons. Microbial counting in experiment III from the antibiotic treatment or the control group was compared with Mann–Whitney *U* test or independent *t* test. All the statistical analyses were carried out in IBM SPSS 20.0 (International Business Machines Corp., Armonk, New York, USA). Figures were produced using SigmaPlot 14.0 (Systat Software Inc., San Jose, California, USA).

## Results

### Result I: *B. bassiana* Did Not Infect and Colonize on *D. antiqua* Larvae Due to Associated Microbiota

**Figure 1:**
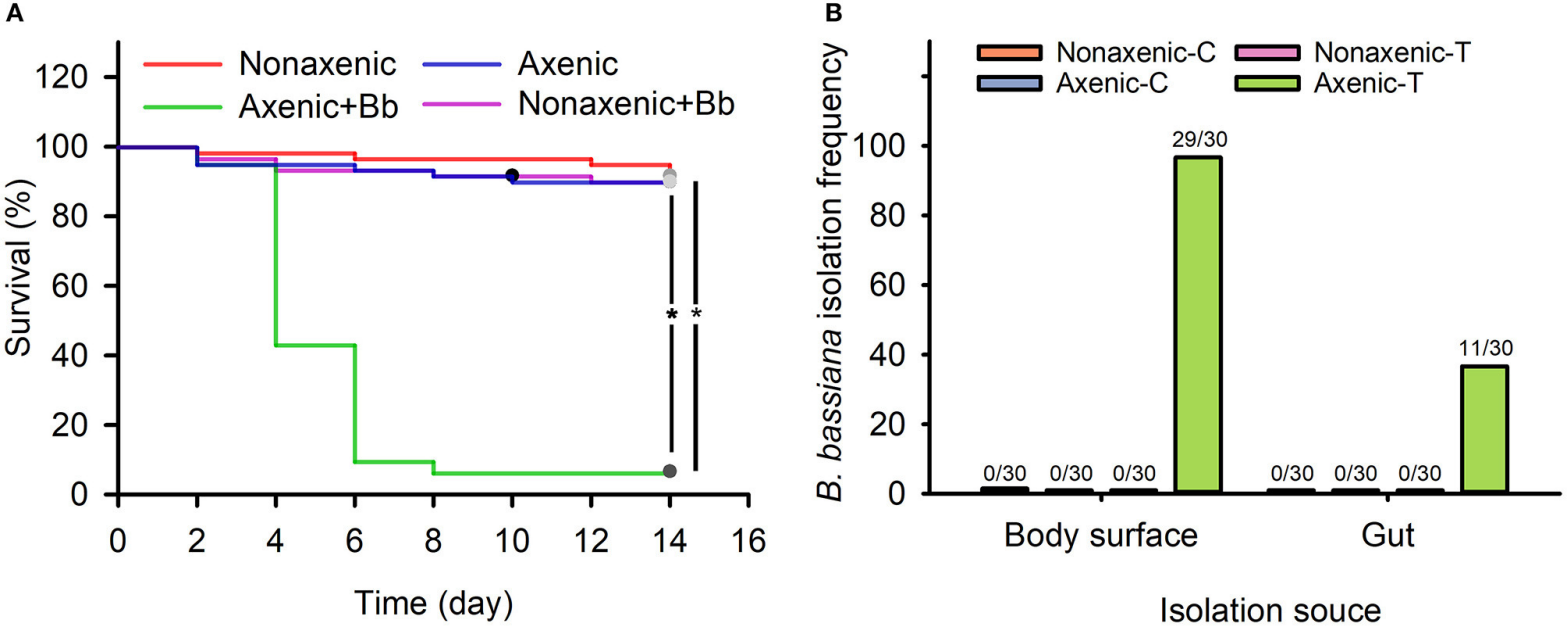
Associated microbiota inhibited *B. bassiana* infection of *D. antiqua* larvae and colonization of the fungus on *D. antiqua* larvae. Survival of axenic and non-axenic *D. antiqua* larvae treated with *B. bassiana* BB1101 **(A)**. The red, blue, green, and pink lines refer to survival of non-axenic larvae without *B. bassiana* treatment, axenic larvae without *B. bassiana* treatment, non-axenic larvae treated with *B. bassiana*, and axenic larvae treated with *B. bassiana*, respectively. *B. bassiana* isolation frequency **(B)** from non-axenic larvae treated with phosphate-buffered saline (PBS) (non-axenic-C), axenic larvae treated with PBS (axenic-C), non-axenic larvae treated with *B. bassiana* conidial suspension (non-axenic-T), and axenic larvae treated with *B. bassiana* conidial suspension (axenic-T). Larval survival was estimated with Kaplan–Meier analysis (*n* = 30, one representative experiment of three, log-rank test, α = 0.05). Asterisk (^*^) denotes a significant difference between the two connected lines.

Compared to untreated larvae, survival of non-axenic larvae was not significantly affected by *B. bassiana* treatment (Figure 1A, χ^2^ = 0.133, df = 1, *p* = 0.977). However, as compared to untreated larvae, survival of axenic larvae significantly reduced due to *B. bassiana* treatment (Figure 1A, χ^2^ = 86.000, df = 1, *p* < 0.001). Specifically, treated axenic larval survival was 6.7%, which was significantly lower as compared to untreated axenic larval survival, which was 90.0% (Figure 1A, χ^2^ = 86.014, df = 1, *p* < 0.001).

No *B. bassiana* strains were isolated from untreated non-axenic and axenic *D. antiqua* larvae (Figure 1B). Furthermore, no *B. bassiana* strains were isolated from non-axenic larvae treated with *B. bassiana* conidial suspension. *B. bassiana* strains were isolated from body surface samples (29/30) and gut samples (11/30) of axenic larvae treated with *B. bassiana* (Figure 1B).

### Result II: Microbiota of *D. antiqua* Larvae Was Stable Under the Effects of *B. bassiana*

In total, 1,537,245 ITS and 1,774,261 16S rRNA sequences were obtained from larval microbiota samples. Using similarity cutoff at 97%, 828,624 ITS sequences were grouped into 27 OTUs; 1,101,710 16S rRNA sequences were grouped into 198 OTUs using similarity cutoff at 97%.

**Figure 2:**
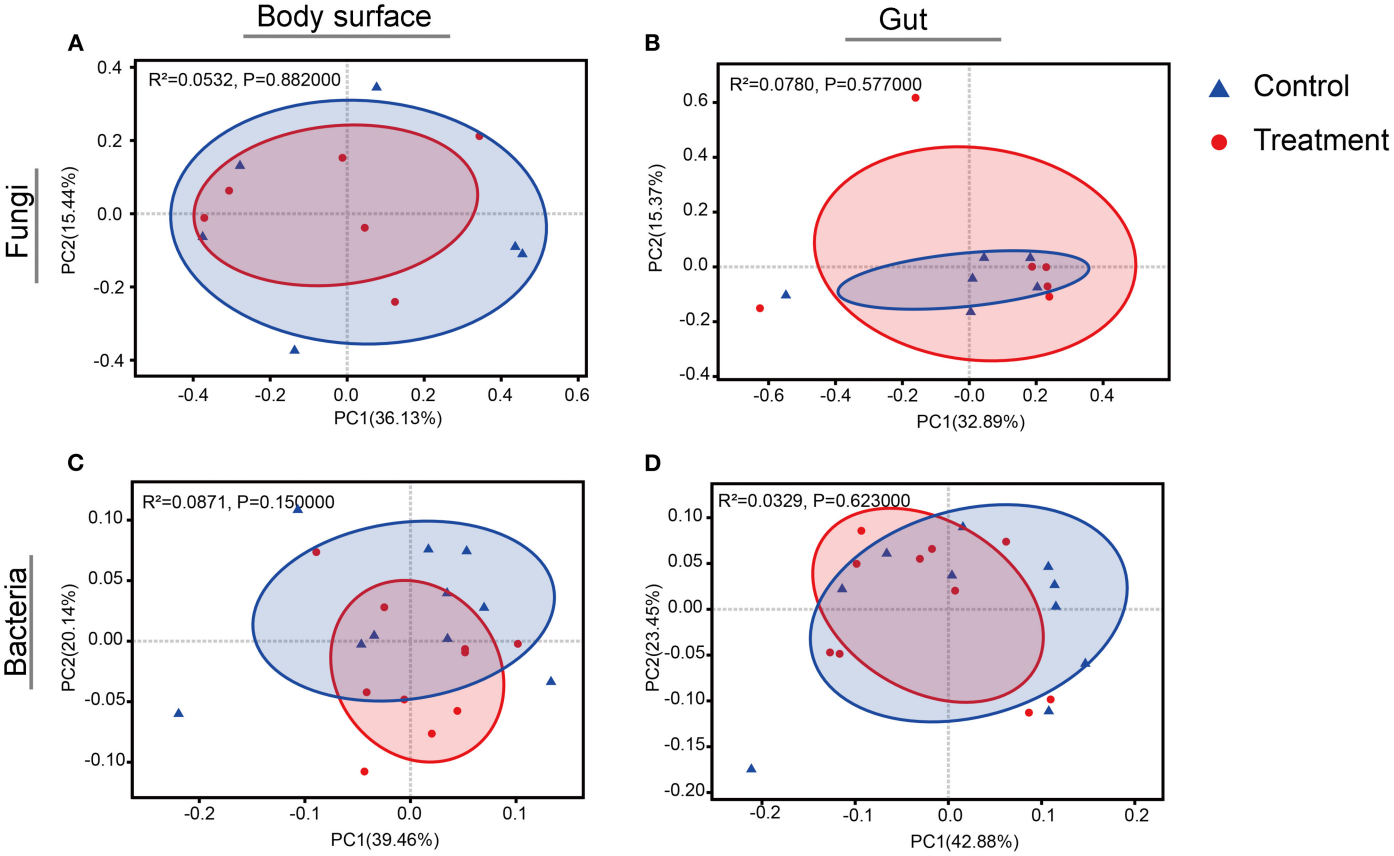
Principal coordinate analysis (PCoA) plots of body surface samples and gut samples for microbial community analysis. **(A–D)** PCoA plots of fungal body surface samples, fungal gut samples, bacterial body surface samples, and bacterial gut samples, respectively. PCoA plots are based on the weighted UniFrac metric for microbial communities. Blue triangles represent body surface or gut samples treated by sterilized PBS (control), and red dots represent samples treated by *B. bassiana* conidial suspension. Significance values refer to analysis of similarity (Adonis, *p* < 0.05) test for differences in community composition between the control and the *B. bassiana*-treated group.

**Figure 3:**
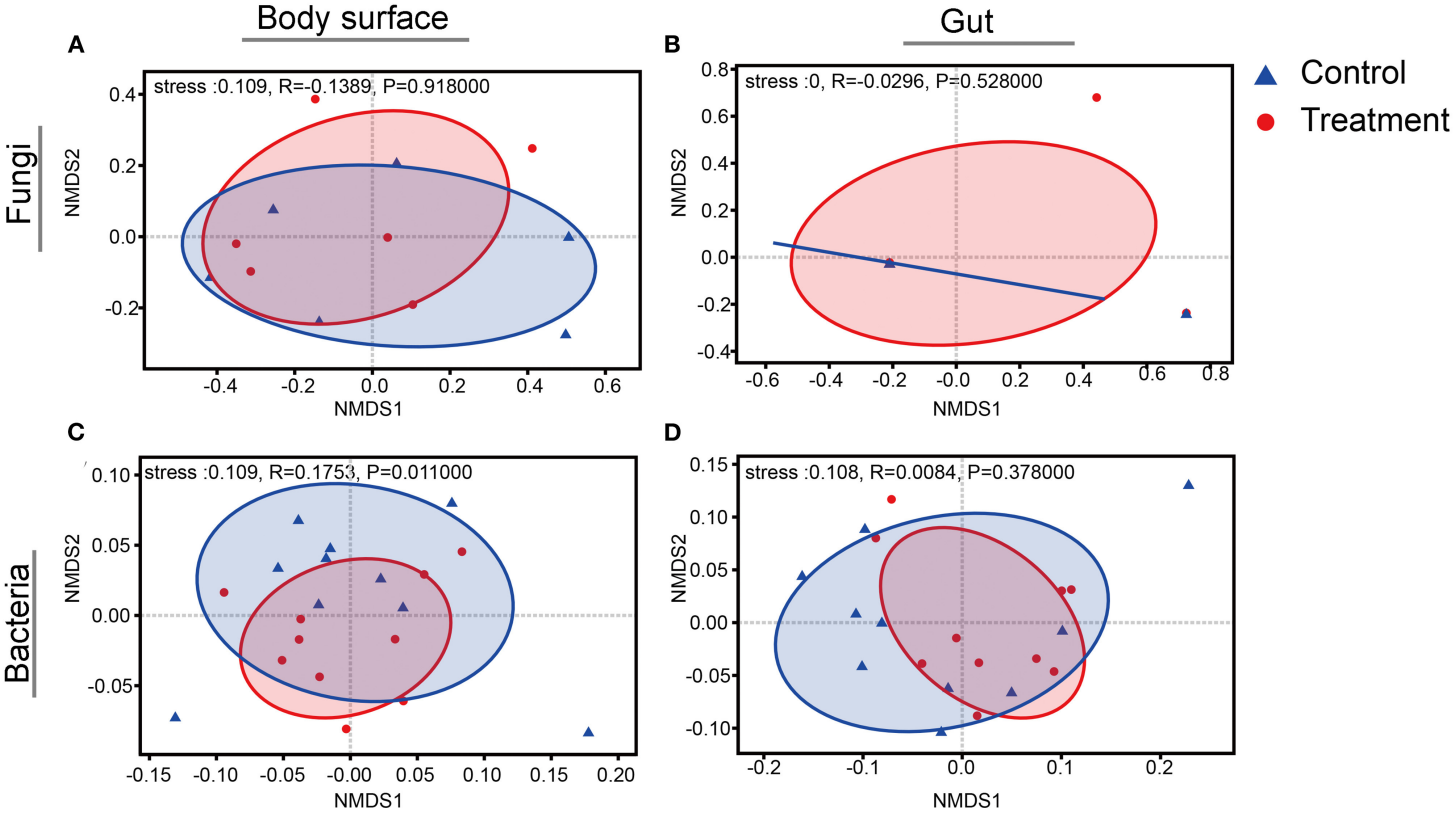
Non-metric multidimensional scaling (NMDS) diagrams of body surface samples and gut samples for microbial community analysis. **(A–D)** NMDS diagrams of fungal body surface samples, fungal gut samples, bacterial body surface samples, and bacterial gut samples, respectively. NMDS diagrams are based on a Bray–Curtis distance matrix for microbial communities that consisted of operational taxonomic units (OTUs) (97% similarity level). Blue triangles represent body surface or gut samples treated by PBS (control), and red dots represent samples treated by *B. bassiana*. Significance values refer to analysis of similarity (ANOSIM, *p* < 0.05) test for differences in community composition between the control group and the *B. bassiana*-treated group.

Rarefaction curves for sample sequences from both the body surface and the gut groups almost reached equilibrium (Supplementary Figure 1). For both the fungal and bacterial microbiota, neither the body surface nor the gut samples showed significant differences for alpha diversity indices between the control and the *B. bassiana*-treated groups (Table 1, Student's *t*-test, *p* > 0.05). Considering relative abundances of OTUs, PCoA showed similar distributions. In the fungal microbiota, neither the larval body surface samples nor the gut samples from the control and the *B. bassiana*-treated groups cluster independently or distinctly from each other (Figures 2A,B). In the bacterial microbiota, neither the larval body surface samples nor the gut samples from the control and the *B. bassiana*-treated groups show a trend of independent or distinct clustering from each other (Figures 2C,D). In the fungal microbiota, NDMS diagram using the Bray–Curtis similarity metric showed that body surface (Figure 3A) and gut (Figure 3B) samples from the control group and the *B. bassiana*-treated group did not cluster independently or distinctly from each other. However, in the bacterial microbiota, the NDMS diagram using the Bray–Curtis similarity metric showed that body surface (Figure 3C) samples from the control and the *B. bassiana*-treated groups clustered independently and distinctly from each other, while gut (Figure 3D) samples from the control and the *B. bassiana*-treated groups did not cluster independently or distinctly from each other.

**Table 1:** Diversity indices of fungal and bacterial community from body surface and gut samples of *D. antiqua* larvae.

**Samples**		**Index**	**Control^a^**	***B. bassiana*** **treated^b^**	***P*** **value^c^**	***Q*** **value**
**Fungal**	**Body surface**	Number of OTUs	12.16 ± 1.83	15.16 ± 3.48	0.09	0.31
		ACE diversity	14.00 ± 4.21	13.93 ± 9.22	0.99	1.00
		Chao1 diversity	12.33 ± 1.86	15.33 ± 3.66	0.10	0.31
		Shannon diversity	1.72 ± 0.33	1.73 ± 0.34	0.97	1.00
		Simpson's diversity	0.24 ± 0.08	0.27 ± 0.10	0.69	1.00
	**Gut**	Number of OTUs	12.66 ± 2.25	15.00 ± 4.98	0.48	0.41
		ACE diversity	9.33 ± 7.25	11.16 ± 8.86	0.70	0.52
		Chao1 diversity	12.66 ± 2.25	15.00 ± 4.98	0.48	0.41
		Shannon diversity	1.27 ± 0.34	1.53 ± 0.44	0.48	0.52
		Simpson's diversity	0.45 ± 0.15	0.34 ± 0.14	0.48	0.41
**Bacterial**	**Body surface**	Number of OTUs	18.90 ± 2.23	22.70 ± 10.47	0.28	0.41
		ACE diversity	22.99 ± 6.30	26.23 ± 11.21	0.44	0.52
		Chao1 diversity	20.60 ± 4.68	24.98 ± 10.64	0.25	0.41
		Shannon diversity	1.46 ± 0.16	1.50 ± 0.10	0.52	0.52
		Simpson's diversity	0.32 ± 0.04	0.30 ± 0.03	0.16	0.41
	**Gut**	Number of OTUs	54.70 ± 33.94	66.60 ± 37.92	0.47	0.90
		ACE diversity	97.30 ± 47.38	99.83 ± 41.67	0.64	0.90
		Chao1 diversity	78.64 ± 38.03	86.91 ± 40.50	0.83	0.90
		Shannon diversity	0.69 ± 0.46	0.65 ± 0.23	0.87	0.90
		Simpson's diversity	0.70 ± 0.16	0.71 ± 0.12	0.90	0.90

a *“Control” represents samples from non-axenic D. antiqua larvae which were treated by PBS*.

b *“B. bassiana treated” represents samples from non-axenic D. antiqua larvae which were treated by B. bassiana BB1101 conidial suspension*.

c *Statistical tests were performed using Student's t test*.

**Figure 4:**
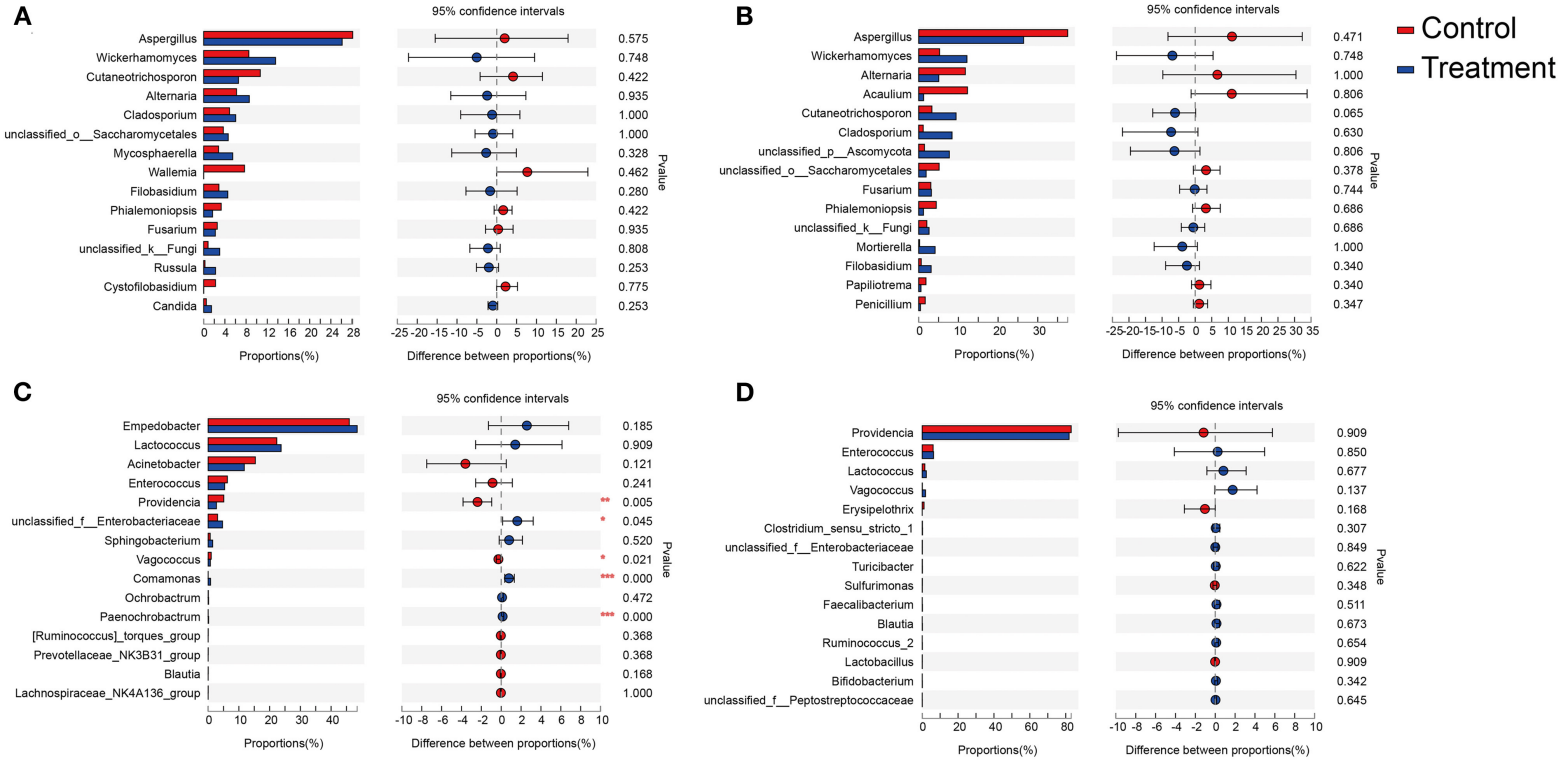
Relative abundances of the top 15 fungal and bacterial genera in body surface samples and gut samples of *D. antiqua* larvae. **(A–D)** The relative abundance comparison of fungal genera from body surface, fungal genera from gut, bacterial genera from body surface, and bacterial genera from gut, respectively. Student's *t-*test was used to evaluate the significance of differences between the control group and *B. bassiana-*treated group. “Control” represents samples from *D. antiqua* larvae treated with sterilized PBS, and “treatment” represents samples from *D. antiqua* larvae treated with *B. bassiana* conidial suspension. Asterisk (^*^) denotes a significant difference between the two groups (0.01 < *p* ≤ 0.05 marked as ^*^, 0.001 < *p* ≤ 0.01 marked as ^**^, and *p* ≤ 0.001 marked as ^***^).

The top three most abundant fungal genera associated with larva body surface in both the *B. bassiana* and the PBS-treated groups were *Aspergillus* spp., *Wickerhamomyces* spp., and *Cutaneotrichosporon* spp. (Figure 4A). No significant differences were detected among the three genera between the control and the *B. bassiana*-treated groups. The top three most abundant fungal genera in the larval gut for both the *B. bassiana* and the PBS-treated groups were *Aspergillus* spp., *Wickerhamomyces* spp., and *Alternaria* spp. (Figure 4B). No significant differences were detected for the three genera between the control and the *B. bassiana*-treated groups.

The top three most abundant bacterial genera associated with larval body surface in both the *B. bassiana* and the PBS-treated groups were *Empedobacter* spp., *Lactococcus* spp., and *Acinetobacter* spp. (Figure 4C). No significant differences were detected for any of the three genera between the control and the *B. bassiana*-treated groups. The top three most abundant bacterial genera in larval gut for both the *B. bassiana* and the PBS-treated groups were *Providencia* spp., *Enterococcus* spp., and *Lactococcus* spp. (Figure 4D). No significant differences were detected for the three genera between the control and the *B. bassiana*-treated groups.

### Result III: Bacterial Microbiota Inhibited Conidial Germination and Mycelial Growth of *B. bassiana* BB1101

**Figure 5:**
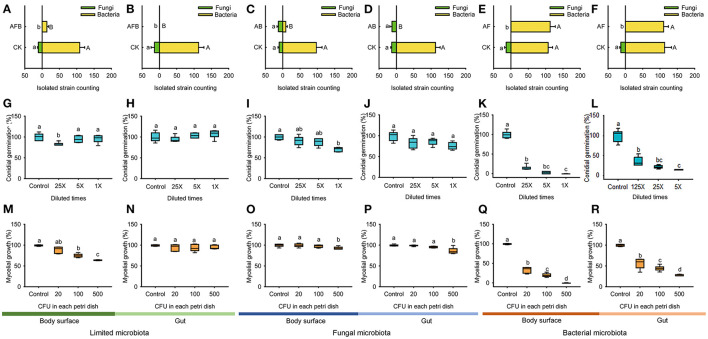
Conidial germination and mycelial growth of *B. bassiana* BB1101 under the effects of limited microbiota, bacterial microbiota, and fungal microbiota. **(A–F)** Bacterial or fungal counting of diluted microbiota suspension from body surface limited microbiota, gut limited microbiota, body surface fungal microbiota, gut fungal microbiota, body surface bacterial microbiota, and gut bacterial microbiota. CK, without antibiotic; AFB, antifungal and antibacterial; AB, antibacterial; AF, antifungal. Different letters beside the bars in **(A–F)** refer to significant difference of microbial counting between the antibiotic treatment group and the control group (capital letters for bacteria, and lowercase letters for fungi. Independent *t*-test or Mann–Whitney *U* test, *p* < 0.05). **(G–L)** Conidial germination of *B. bassiana* BB1101 under the effects of body surface limited microbiota, gut limited microbiota, body surface fungal microbiota, gut fungal microbiota, body surface bacterial microbiota, and gut bacterial microbiota, respectively. **(M–R)** Mycelial growth of *B. bassiana* BB1101 under the effects of body surface limited microbiota, gut limited microbiota, body surface fungal microbiota, gut fungal microbiota, body surface bacterial microbiota, and gut bacterial microbiota, respectively. Different letters above each box refer to significant differences within each set of boxes (one-way or Welch's ANOVA, *p* < 0.05, *n* = 6).

No fungal colonies were isolated from limited microbiota samples of larval body surface and gut, which were significantly different as compared to those in untreated groups (Figure 5A, 11 for larval body surface in the CK, Mann–Whitney *U* test, *Z* = −3.083, *p* < 0.001; Figure 5B, 14.3 for the larval gut in the CK, Mann–Whitney *U* test, *Z* = −3.077, *p* < 0.001). Similarly, only 13.8 bacterial colonies were isolated from the larval body surface of limited microbiota samples, which were significantly lower than those in the untreated group (Figure 5A, 108.3 for larval body surface in the CK, independent *t*-test, *t* = 12.794, *p* = 0.017). No bacterial colonies were isolated from the larval gut of limited microbiota samples, which were significantly different as compared to those in the untreated group (Figure 5B, 112.8 for the larval gut in the CK, Mann–Whitney *U* test, *Z* = −3.083, *p* < 0.001).

Only eight bacterial colonies were isolated from fungal microbiota of larval body surface, and no bacterial colonies were isolated from fungal microbiota of larval gut, which were significantly different as compared to those in untreated groups (Figure 5C, 97.2 for larval body surface in the CK, independent *t*-test, *t* = 12.890, *p* = 0.033; Figure 5D, 112.5 for the larval gut in the CK, Mann–Whitney *U* test, *Z* = −3.077, *p* < 0.001). Moreover, the number of fungal colonies isolated from larval body surface (Figure 5C, independent *t*-test, *t* = −1.21, *p* = 0.652) and gut samples (Figure 5D, independent *t*-test, *t* = 0.238, *p* = 0.241) were not affected significantly by anti-bacterial treatment.

No fungal colonies were isolated from bacterial microbiota samples of the larval body surface and gut, which were significantly different as compared to those in untreated groups (Figure 5E, 13.8 for larval body surface in the CK, Mann–Whitney *U* test, *Z* = −3.077, *p* < 0.001; Figure 5F, 13.3 for the larval gut in the CK, Mann–Whitney *U* test, *Z* = −3.077, *p* < 0.001). Moreover, the number of bacterial colonies isolated from larval body surface (Figure 5E, independent *t*-test, *t* = −0.748, *p* = 0.874) and gut samples (Figure 5F, independent *t*-test, *t* = 0.231, *p* = 0.191) was not affected significantly by antifungal treatment.

Limited microbiota from larval body surface had little effect on conidial germination in *B. bassiana* BB1101 (Figure 5G, one-way ANOVA, *F*_3,20_ = 6.635, *p* = 0.03). However, limited microbiota from the larval gut did not affect the conidial germination (Figure 5H, one-way ANOVA, *F*_3,20_ = 2.300, *p* = 0.108). Fungal microbiota of larval body surface slightly inhibited conidial germination in *B. bassiana* BB1101 (Figure 5I, one-way ANOVA, *F*_3,20_ = 13.784, *p* < 0.001). Specifically, conidial germination was reduced to 91.3, 88.2, and 70.8% relative to control, respectively, under the effect of culture supernatant obtained from larval body surface fungal microbiota diluted25, 5, and 1 times. However, the fungal microbiota of the larval gut did not inhibit conidial germination (Figure 5J, one-way ANOVA, *F*_3,20_ = 2.566, *p* = 0.083). Bacterial microbiota from larval body surface (Figure 5K, one-way ANOVA, *F*_3,20_ = 457.671, *p* < 0.01) and gut (Figure 5L, Welch's ANOVA, *F*_3,8.538_ = 135.503, *p* < 0.01) significantly inhibited conidial germination of *B. bassiana* BB1101. Under the effects of bacterial microbiota from the body surface, conidial germination was suppressed to 15.1, 3.13, and 0.20%, respectively, under the treatment of culture supernatant diluted 25, 5, and 1 times (Figure 5K). Similarly, conidial germination was suppressed to 21.2, 7.9, and 0.5%, respectively, under the treatment of gut bacterial culture supernatant diluted 25, 5, and 1 times (Figure 5L).

Limited microbiota from larval body surface slightly inhibited the mycelial growth of *B. bassiana* BB1101 (Figure 5M, Welch's ANOVA, *F*_3,9.789_ = 73.433, *p* < 0.001). Specifically, mycelial growth was reduced to 89.4, 75.5, and 64.3% relative to control, respectively, under the effect of the microbiota at 20, 100, and 500 CFU/Petri dish. However, limited microbiota from the larval gut did not significantly inhibit mycelial growth (Figure 5N, one-way ANOVA, *F*_3,20_ = 1.583, *p* = 0.220). Fungal microbiota of both larval body surface (Figure 5O, one-way ANOVA, *F*_3,20_ = 4.95, *p* = 0.010) and gut (Figure 5P, one-way ANOVA, *F*_3,20_ = 15.234, *p* < 0.01) slightly inhibited mycelial growth of *B. bassiana* BB1101. Bacterial microbiota from the larval body surface (Figure 5Q, Welch's ANOVA, *F*_3,9.435_ = 572.446, *p* < 0.001) and gut (Figure 5R, Welch's ANOVA, *F*_3,10.062_ = 229.573, *p* < 0.001) significantly inhibited mycelial growth. Under the effects of bacterial microbiota from the body surface, the mycelial growth of *B. bassiana* BB1101was suppressed to 33.9, 20.3, and 0.3%, respectively, under treatment doses of 20, 100, and 500 CFU/Petri dish (Figure 5Q). Similarly, the mycelial growth of the fungus was suppressed to 29.5, 17.8, and 1.1%, respectively, under bacterial gut microbiota treatment doses of 20, 100, and 500 CFU/Petri dish (Figure 5R).

### Result IV: Dominant Bacterial Symbionts Restrained Conidial Germination and Mycelial Growth of *B. bassiana*

**Figure 6:**
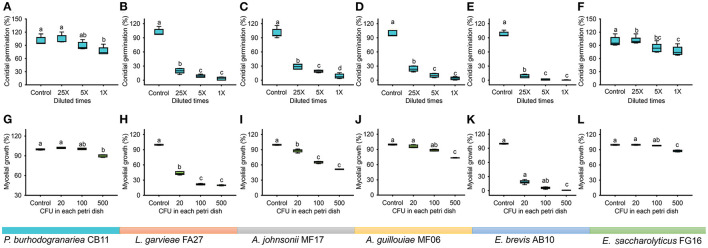
Conidial germination and mycelial growth of *B. bassiana* BB1101 under the effects of dominant bacterial species associated with *D. antiqua* larvae. (**A–F**) Conidial germination of *B. bassiana* BB1101 under the effects of *P. burhodogranariea* CB11, *L. garvieae* FA27, *A. johnsonii* MF17, *A. guillouiae* MF06, *E. brevis* AB10, and *E. saccharolyticus* FG16, respectively. (**G–L**) Mycelial growth of *B. bassiana* BB1101 under the effects of *P. burhodogranariea* CB11, *L. garvieae* FA27, *A. johnsonii* MF17, *A. guillouiae* MF06, *E. brevis* AB10, and *E. saccharolyticus* FG16, respectively. Different letters above each box refer to significant differences within each set of boxes (one-way or Welch's ANOVA, *p* < 0.05, *n* = 6).

Bacterial strains of dominant genera showed significant inhibitory effects in a dose-dependent manner on the conidial germination in *B. bassiana* (Figure 6A, *P. burhodogranariea* CB11, one-way ANOVA, *F*_3,20_ = 11.584, *p* < 0.001; Figure 6B, *L. garvieae* FA27, Welch's ANOVA, *F*_3,10.595_ = 416.628, *p* < 0.001; Figure 6C, *A. johnsonii* MF17, Welch's ANOVA, *F*_3,10.195_ = 327.030, *p* < 0.001; Figure 6D, *A. guillouiae* MF06, Welch's ANOVA, *F*_3,10.515_ = 364.944, *p* < 0.001; Figure 6E, *E. brevis* AB10, Welch's ANOVA, *F*_3,8.816_ = 627.251, *p* < 0.001; Figure 6F, *E. saccharolyticus* FG16, one-way ANOVA, *F*_3,20_ = 10.564, *p* = 0.002). Specifically, under the effect of *L. garvieae* FA27, *A. johnsonii* MF17, *A. guillouiae* MF06, and *E. brevis* AB10, conidial germination in *B. bassiana* BB1101 was sharply inhibited to less than 30% as compared to the control group even at bacterial culture supernatant dilution to 25 times. However, *P. burhodogranariea* CB11 and *E. saccharolyticus* FG16 have little inhibitory effects on the conidial germination.

Mycelial growth of *B. bassiana* BB1101 was also significantly inhibited by these bacteria (Figure 6G, *P. burhodogranariea* CB11, one-way ANOVA, *F*_3,20_ = 134.785, *p* < 0.001; Figure 6H, *L. garvieae* FA27, Welch's ANOVA, *F*_3,10.670_ = 3,219.676, *p* < 0.001; Figure 6I, *A. johnsonii* MF17, Welch's ANOVA, *F*_3,9.682_ = 1,025.624, *p* < 0.001; Figure 6J, *A. guillouiae* MF06, Welch's ANOVA, *F*_3,9.703_ = 318.465, *p* < 0.001; Figure 6K, *E. brevis* AB10, Welch's ANOVA, *F*_3,8.837_ = 3,131.672, *p* < 0.001; Figure 6L, *E. saccharolyticus* FG16, Welch's ANOVA, *F*_3,10.427_ = 217.658, *p* < 0.001). Specifically, *L. garvieae* FA27 and *E. brevis* AB10 showed greater inhibition of mycelial growth. Under the effect of these three bacteria, the mycelial growth of *B. bassiana* BB1101 was sharply inhibited to 20–40% as compared to the control group even under treatment with 20 CFU/ml. Relatively, *P. burhodogranariea* CB11, *A. johnsonii* MF17, *A. guillouiae* MF06, and *E. saccharolyticus* FG16 showed lesser inhibition on mycelial growth of *B. bassiana* BB1101.

## Discussion

In this work, to the best of our knowledge, the response of the associated microbiota in *D. antiqua* larvae to *B. bassiana* was determined for the first time. During the invasion of *B. bassiana* into the insect–microbe symbiosis, the fungus could not infect *D. antiqua* larvae due to the associated microbiota (Figure 1A). It could not colonize the larvae (Figure 1B). For both the bacterial and fungal communities, diversity indices were not significantly affected (Table 1). Moreover, for both the bacterial or fungal communities, results of PCoA (Figure 2) showed no significant differences in the microbiota of *B. bassiana* with or without treatment; however, NMDS (Figure 3) showed slight differences between the microbiota between the treated and untreated larvae. Additionally, the relative abundances of dominant fungal and bacterial genera both in the larval body surface and gut were not significantly affected by *B. bassiana* (Figure 4). These results indicated that microbiota associated with *D. antiqua* larvae was stable after *B. bassiana* treatment. In other words, *B. bassiana* showed no significant effects on the associated microbiota. A reasonable explanation for this could be the blockage of *B. bassiana* conidia addition into the microbial community. Further results showed that culturable fungus microbiota had little effects on inhibition on conidial germination and mycelial growth of *B. bassiana*, while culturable bacterial microbiota almost completely inhibited conidial germination and mycelial growth of *B. bassiana* (Figure 5). Thus, conidia addition into the symbiotic system was blocked by the culturable bacterial microbiota. Consequently, the fungal and bacterial communities were not affected. Subsequent tests showed that strains from dominant bacterial genera including *L. garvieae* FA27, *A. johnsonii* MF17, *A. guillouiae* MF06, and *E. brevis* AB10 significantly inhibited conidial germination and mycelial growth of *B. bassiana* (Figure 6). Taken together, bacterial microbiota associated with *D. antiqua* larva blocked *B. bassiana* conidia and consequently stabilized the associated microbiota.

One interesting phenomenon is that bacterial microbiota shows significant inhibition on conidial germination and mycelial growth of *B. bassiana* BB1101, while fungal microbiota does not. Actually, the interaction between bacteria and fungi has been widely investigated (Wargo and Hogan, 2006; Kobayashi and Crouch, 2009). It seems that the bacterial inhibition on fungi is a common phenomenon in various symbiotic systems (Chevrette et al., 2019). For example, bark beetle-associated bacteria inhibit the colonization of ophiostomatoid fungi and consequently reduce the consumption of saccharide in pine tree phloem (Zhou et al., 2016). Similar results have also been reported in multiple animals such as beetles (Heise et al., 2019), bees (Arredondo et al., 2018; Wang et al., 2018), and cockroaches (Zhang et al., 2013). As it has been reported, bacteria can inhibit fungal laccase activity, and some bacterial volatiles show excellent antifungal activity (Mackie and Wheatley, 1999), which may explain the antifungal activity of the bacterial strains associated with *D. antiqua* larvae. Compared to the efficient antifungal activity of associated bacteria, antifungal fungal species are scarcely reported.

Another interesting observed phenomenon was the relatively higher anti-*B. bassiana* effect of bacterial microbiota associated with larval body surface than in the gut (Figures 5K,L,Q,R). This may result from differences in species composition between the larval body surface and gut microbiota. Dominant bacterial species of microbiota on larval body surface belong to genera including *Empedobacter, Lactococcus*, and *Acinetobacter* (Figure 4C), while dominant bacterial species of microbiota inside larval gut belong to genera including *Providencia* and *Enterococcus* (Figure 5D). In addition, bacteria such as *L. garvieae, A. johnsonii, A. guillouiae*, and *E. brevis* showed significant inhibitory effects on conidial germination and mycelial growth of *B. bassiana*, while bacterial strains including *P. burhodogranariea* and *E. saccharolyticus* showed little inhibitory effects on conidial germination and mycelial growth of *B. bassiana*. Thus, there was differential inhibition on the larval body surface and gut microbiota. *B. bassiana* usually infects the insect through its cuticle (Wei et al., 2017); thus, the high inhibition on *D. antiqua* larval body surface microbiota may contribute to the protection of the insect from *B. bassiana* infection. Conversely, due to limited inhibitory effect in the gut as compared to that of larval body surface microbiota, *D. antiqua* larval gut seems to be a relatively easier route of infection by *B. bassiana*. However, additional factors including pH, oxygen, and immune responses (Zhang et al., 2017) may also affect the colonization of the fungus inside the insect gut, and these need to be investigated in future studies.

In previous studies that report the individual bacterial species associated with *D. antiqua* larvae, frequently isolated bacteria including *Citrobacter freundii, Enterobacter ludwigii, Pseudomonas protegens, Serratia plymuthica, Sphingobacterium faecium*, and *Stenotrophomonas maltophilia* showed excellent anti-*B. bassiana* activity (Zhou et al., 2019). With a culture-dependent method that is limited by nutrients of culture medium and microbial growth character, the full scale of bacterial diversity associated with *D. antiqua* may not be reflected as in the previous studies. Differently, the bacterial diversity of *D. antiqua* was investigated using culture-independent methods, i.e., pyrosequencing in this study. Subsequently, the dominant bacterial genera were confirmed. As the current pyrosequencing could not annotate sequence at the species level, *E. brevis, L. garvieae, A. johnsonii*, and *A. guillouiae* were isolated and selected as the representative dominant species. These bacteria showed excellent antifungal activity. Taken together, the results indicated that the majority of the associated bacteria could protect the larvae from *B. bassiana* infection. Compared to the anti-pathogen effects of some specialized symbionts associated with animal species such as the wasp (Engl et al., 2018), bees (Arredondo et al., 2018), and attelabid weevil (Wang et al., 2015), *D. antiqua* larvae seemed to harbor more microbial species for their protection. Two possible mechanisms may underlie this phenomenon. First, given that *B. bassiana* could hardly infect *D. antiqua* larvae in fields, they may be an unspecialized entomopathogen of *D. antiqua*. Thus, the anti-pathogen effect of associated bacteria could function as a primary defense of the insect host against pathogens. Second, the dominant bacterial strains in this study and the selected bacterial strains in previous studies may share common active metabolites. If the phylogenetic relationships of these bacterial strains are taken into consideration, common primary metabolites produced by bacteria from various taxonomic clades instead of specialized secondary metabolites may be excellent for anti-*B. bassiana* activity. For example, antifungal effects of some organic acids including phenyl lactic acid (Mu et al., 2012) and indoleacetic acid (Yu et al., 2009) produced during amino acid metabolism are reported. However, this assumption needs further metabonomic analysis of the bacterial strains. In addition to focusing on the interaction between the insect-associated bacteria and *B. bassiana*, the direct interaction between the insect and entomopathogen was also investigated. By investigating fly immunity, a previous study suggests that insects can encode and secrete various antimicrobial peptides against pathogens (Xia et al., 2021). As for *D. antiqua*, if this insect could also produce antimicrobial peptides against *B. bassiana* remains unknown. Further, to what extent associated bacteria or the immunity contribute to the inhibition of *B. bassiana* needs to be investigated in the future.

In this study, the stability of microbiota associated with *D. antiqua* by blocking pathogens was elucidated. Subsequent work should focus on deciphering the effect of microbiota without the dominant species on *B. bassiana* infection in axenic *D. antiqua* larvae. Besides, whether the variation of the microbiota can influence the resistance of non-axenic *D. antiqua* larvae against *B. bassiana* infection also needs to be investigated. One feasible way to test it is by constructing an artificial microbiota with individual microbial strains and subsequently determining its functions; such studies have been reported for the rhizosphere microbiome (Liu et al., 2019).

In complex symbiosis formed by an animal host and its associated microbes, the interaction between the microbiota and the immune system of the host along with the environment (Coyte et al., 2015) influences the stability of the associated microbiota of the host essential for the maintenance of the symbiosis. Stability includes both resilience and resistance (Allison and Martiny, 2008). Resilience is defined as the composition and function of an ecosystem rebounding to the original state or close to the original state after a perturbation, and resistance is when the ecosystem remains unchanged in response to a disturbance (Allison and Martiny, 2008). In this study, the interaction between the pathogen *B. bassiana* and the associated microbiota, the pathogen, i.e., the factor that might disrupt the stability of the microbiota, met the resistance of the microbiota. Consequently, the factor was completely blocked by the microbiota, which was a relatively economic way of keeping the microbiota stable. The block of the pathogen by the microbiota associated with *D. antiqua* larvae could also avoid the activation of the host immunity system (Xu et al., 2018b), which could be a result of a long time of co-evolution. This study sheds light on the function of animal-associated microbiota and deepened the understanding of animal–microbe co-evolution. Pathogens, especially some fungi like *B*. *bassiana*, have been widely used as pest biocontrol agents. By elucidating the role of associated microbiota during pathogen infection, this work could provide novel strategies for the development of efficient biocontrol agents, particularly for entomopathogens that cannot be inhibited by the associated microbiota.

## Data Availability Statement

The datasets presented in this study can be found in online repositories. The names of the repository/repositories and accession number(s) can be found at: https://www.ncbi.nlm.nih.gov/bioproject/PRJNA756127/.

## Author Contributions

FZ was responsible for the conceptualization, methodology, software, data curation, and writing—original draft preparation. YG was responsible for visualization and investigation. ML was responsible for the investigation. LX and XW were responsible for writing—reviewing and editing. XiaoZ was responsible for the investigation. XinZ was responsible for supervision, software, validation, and writing—reviewing and editing. All authors contributed to the article and approved the submitted version.

## Funding

This work was funded by the National Natural Science Foundation of China (31700426 and 31901928) and the Key Research and Development Program of Shandong Province (2019GSF109056, 2019JZZY020610, and 2019GSF109012).

## Conflict of Interest

The authors declare that the research was conducted in the absence of any commercial or financial relationships that could be construed as a potential conflict of interest.

## Publisher's Note

All claims expressed in this article are solely those of the authors and do not necessarily represent those of their affiliated organizations, or those of the publisher, the editors and the reviewers. Any product that may be evaluated in this article, or claim that may be made by its manufacturer, is not guaranteed or endorsed by the publisher.
