# Supplementary material for: Bacterial Inhibition on Beauveria bassiana Contributes to Microbiota Stability in Delia antiqua
Source: Front Microbiol. 2021 Oct 6;12:710800. doi: 10.3389/fmicb.2021.710800 (PMC8527029; doi:10.3389/fmicb.2021.710800)
Supplement: Supplementary file 1 [file Data_Sheet_1.docx]

Supplementary Material

# Supplementary Figures and Tables

## Supplementary Figures

**
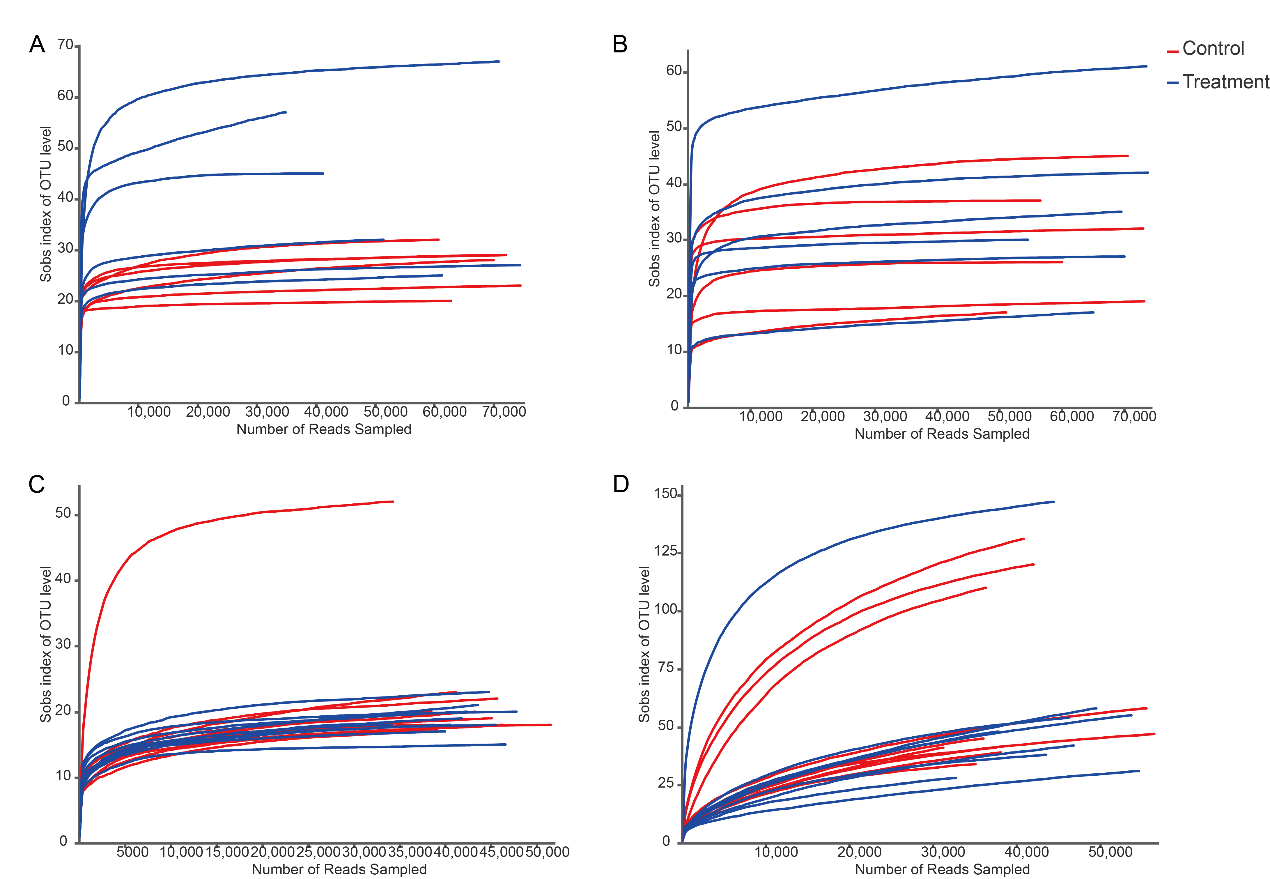
**

**Supplementary Figure 1.** Rarefaction curves of ITS sequencing with samples from both the body surface group (**A**) and the gut group (**B**), and rarefaction curves of 16S rRNA sequencing with samples from both the body surface group (**C**) and the gut group (**D**).

## Supplementary Tables

**Table S1 Artificial diets for axenic *D. antiqua* larvae^*^**

| Ingredients | Dosage (g/750 mL H_2_O) | Producers |
| --- | --- | --- |
| Rat feed | 48 | Biotech-hd biotechnology co. LTD |
| Defatted soybean powder | 56 | Solarbio Life Sciences |
| Cellulose powder | 40 | Herbalife International of America, Inc. |
| Sucrose | 16 | Solarbio Life Sciences |
| Agar | 16 | Solarbio Life Sciences |
| Yeast extract | 16 | Solarbio Life Sciences |
| Methylparaben | 3 | Merck KGaA, Darmstadt, Germany |
| Chloramphenicol | 1.5 | Merck KGaA, Darmstadt, Germany |
| Penicillin G sodium salt | 1,5 | Solarbio Life Sciences |
| Neomycin sulphate | 1.5 | Merck KGaA, Darmstadt, Germany |
| Choline chloride | 1.2 | Merck KGaA, Darmstadt, Germany |
| Vitamin C | 1.2 | Solarbio Life Sciences |

* Water, rat feed, defatted soybean powder, cellulose powder, sucrose, agar, and yeast extract were mixed in water and sterilized at 121 °C for 20 min, and methylparaben, chloramphenicol (dissolved in ethanol), penicillin G sodium salt, neomycin sulphate, choline chloride and vitamin C were added into the above sterilized mixture after sterilization.

# Supplementary Methods

**2.1 Isolation and identification of bacterial symbionts associated with *D. antiqua* larvae**

*D. antiqua* larvae were collected from garlic fields in Fanzhen, Taian City, China. Approximately 7 garlic plants that had been attacked (and were at least 300 m from one another) were sampled for *D. antiqua* larvae. In total, 9 *D. antiqua* 3^rd^ instar larvae were collected. To isolate bacteria from the larval body surface, each larva was immersed in 200 μL of 10% PBS in a 2.0-mL centrifuge tube, and the sample was sonicated for 1 min and vortexed for 10 sec. then, 100 μL of the diluted suspension was spread onto Luria-Bertani agar plates containing nystatin (40 mg/L) and cycloheximide (0.5 mg/L). To isolate bacteria from the larval guts, individual guts were obtained by dissecting the surface-sterilized larvae. Gut samples were crushed, diluted and spread onto LB agar plates. All plates were incubated at 28 °C for 12-48 h. Colonies from each plate were counted, and those with different sizes, colors, thicknesses, transparencies, and textures were selected and streaked on LB agar plates for purification. Pure cultures were grouped basing on colony morphology. Bacterial strains isolated from at least two samples in each group were identified by 16S rDNA sequencing. Bacterial DNA was extracted with a PureLink™ Microbiome DNA Purification Kit (Thermo Scientific), and the 16S rDNA gene was amplified with the following primers:

1492 R (5’-GGCTCGAGCGGCCGCCCGGGTTACCTTGTTACGACTT-3’) and

8F (5’-GCGGATCCGCGGCCGCTGCAGAGTTTGATCCTGGCTCAG-3’).

The PCR conditions were as follows: 5 min at 94°C, 35 cycles of 30 sec at 94°C, 30 sec at 51°C, and 1 min 30 sec at 72°C, with a final extension for 10 min at 72°C. PCR products were sequenced in two directions by an ABI 3730XL DNA analyzer (Applied Biosystems, USA) with the same primers, i.e., 1492R and 8F. Sequences were manually assembled and edited with MEGA X. All obtained 16S rDNA sequences were blasted online with the EzBioCloud database (http: //www. eztaxon-e.ezcloud.net/) and BLAST search (http://blast.ncbi.nlm.nih.gov/Blast.cgi), and strains in this study were identified according to the blast result.
